# A mini-review of the role of condensin in human nervous system diseases

**DOI:** 10.3389/fnmol.2022.889796

**Published:** 2022-08-04

**Authors:** Du Pang, Shengping Yu, Xuejun Yang

**Affiliations:** ^1^Department of Neurosurgery, Tianjin Medical University General Hospital, Tianjin Neurological Institute, Tianjin, China; ^2^Department of Neurosurgery, Beijing Tsinghua Changgung Hospital, Affiliated Hospital of Tsinghua University, Beijing, China

**Keywords:** condensin, mitosis, chromosome, 3D chromatin architecture, nervous system diseases

## Abstract

Mitosis and meiosis are crucial life activities that transmit eukaryotic genetic information to progeny in a stable and orderly manner. The formation and appearance of chromosomes, which are derived from chromatin, are the preconditions and signs of mitosis. When entering mitosis, interphase loose chromatin is highly spiralized and folded to form compact chromosomes. In recent years, it has been found that in addition to the well-known DNA, histones, and topoisomerase, a large protein complex called condensin plays an important role in the process of chromosome formation. Numerous studies have shown that the abnormal function of condensin can lead to incomplete or excessive concentration of chromatin, as well as disorder of genome organization process, abnormal transmission of genetic information, and ultimately lead to various diseases of individual, especially in nervous system diseases. In this review, the biological function of condensin and the potential pathogenic mechanism of condensin in nervous system diseases are briefly summarized. Therefore, the investigation of these mechanisms makes a significant contribution to the understanding of those related diseases and provides new ideas for clinical treatments.

## Introduction

Mitosis and meiosis are important life activities that transmit eukaryotic genetic information to progeny in a stable and orderly manner, which are of great significance for maintaining the normal growth and development of individuals and ensuring the continuity and stability of species. The formation and appearance of chromosomes are the premise and symbol of mitosis, and also the guarantee of accurate inheritance of genetic material to the offspring. Chromosome comes from chromatin, which is a complex of DNA, histones, non-histones, and a small amount of RNA (Luger et al., 1997). Chromatin is loosely expanded in the interphase and highly spiralized and folded to form a compact chromosome as it enters mitosis (Vagnarelli, 2012). It was found that although nucleosomes are the basic unit of chromatin packaging, some functional proteins such as topoisomerase, CTCF, cohesion, and condensin play their roles in this packaging process in addition to DNA and histones (Pommier et al., 2022). Condensins, assisting in the construction of 3D chromatin architecture, play their functions in chromosome condensation and segregation during mitosis by participating in the formation of chromatin loops and topologically associated domains (TAD; Kalitsis et al., 2017; Tanizawa et al., 2017). Recent studies have also confirmed that 3D chromatin architecture changes dynamically and maintains homeostasis during ontogeny, which plays an important role in DNA replication, gene expression regulation, cell differentiation, and development (Ke et al., 2017; Zheng and Xie, 2019). Peculiarly, these changes in 3D chromatin architecture are essential for the division of neural stem cells, the maturation of post-mitotic neurons, neurodevelopment, and neurodegeneration, in which the normal function of condensin is indispensable (Nishide and Hirano, 2014; Davis et al., 2018; Hassan et al., 2020; Hu et al., 2021). On the contrary, the abnormal function of condensin can influence the 3D chromatin folding leading to the disorder of gene expression regulation, and ultimately cause various diseases especially nervous system diseases (Norton and Phillips-Cremins, 2017; Li et al., 2018). Thus in this mini-review, we briefly summarize the biological function of condensins and their potential pathogenesis in nervous system diseases, including nervous system developmental disorders, nervous system tumors, and Alzheimer’s disease, which may provide new understanding and treatment ideas for these diseases.

## The Biological Function of Condensin

**Figure 1:**
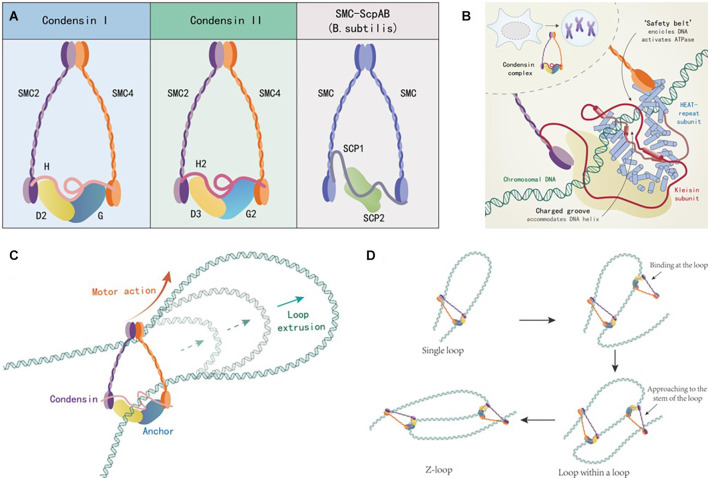
**(A)** Structural schematic diagram of condensing. **(B)** “safety belt” structure and DNA anchoring. **(C)** Mechanism of DNA loop formation promoted by condensins. **(D)** Mechanism of multiple condensins promoting DNA Z-loop formation.

Condensin is a large protein complex composed of five protein subunits, which is highly conserved in both eukaryotes (fungi, vertebrates) and prokaryotes (*Bacillus subtilis*) during the evolutionary process of species. Up to now, three types of condensin have been found. Condensin I and II exist in humans and most other eukaryotes. The third type of condensin was found in Bacillus subtilis. Condensin I and II have two same structural maintenance of chromosomes (SMC) subunits 2 and 4 (SMC2/4), while three different non-SMC regulatory subunits which are a kleisin subunit (NCAPH and NCAPH2) and a pair of HEAT subunits (NCAPD2/G and NCAPD3/G2). The third type of condensin, in the form of SMC-ScpA/B, consists of two identical SMC subunits, two identical ScpB subunits, and one kleisin subunit (ScpA). Therein the hinge domains of these two SMC subunits are combined with each other, while the head domains are combined with their regulatory subunits respectively. Two SMC subunits form an “∧” shaped dimer. Then the kleisin subunit connects with the head domain of the dimer asymmetrically in a band shape. The HEAT subunits bind to the central domain of kleisin subunit. There is an ATP binding domain at the head domain, which is used to regulate the contact and separation between dimer and regulatory subunit, as shown in Figure 1A (Hirano, 2016). Condensin can be attached to dsDNA as ATP hydrolysis-dependent molecular motor and move along dsDNA as needed (Terakawa et al., 2017). Otherwise, the kleisin subunit and the HEAT subunits together constitute the groove domain for recognizing dsDNA, in which the “safety belt” structure (red part) formed by kleisin subunit is the key for recognizing and anchoring dsDNA, as shown in Figure 1B (Kschonsak et al., 2017). So, one end of the condensin is firmly anchored to the dsDNA, and the other end is attached to the dsDNA and moves along the dsDNA in a certain direction, prompting the linear dsDNA segment to form a dsDNA loop, as shown in Figure 1C (Ganji et al., 2018). When multiple condensins are attached to the same linear dsDNA segment, they mutually reinforce rather than interfere with each other. Finally, the single linear dsDNA segment is folded into a Z-loop composed of three parallel connected dsDNA segments, and the linear dsDNA is further compressed, as shown in Figure 1D (Kim et al., 2020). Condensins mediate in loop extrusion in this way, as a means for 3D genome organization. It is worth mentioning that the distribution of condensin I and II on chromosomes is different, as well as their relative concentrations and ratios *in vivo* by which the chromosome shapes are determined. First, both of them take different effects in chromosome condensation and segregation during mitosis: condensin I mainly promotes the longitudinal compression of chromosomes, while condensin II mainly serves the axial compression. Second, condensin I is sequestered in the cytoplasm during interphase and gains access to chromosomes only after the nuclear envelope breaks down in prometaphase, while condensin II localizes to the nucleus during interphase and prophase and participates in an early stage of chromosome condensation within the prophase nucleus (Ono et al., 2003, 2004; Shintomi and Hirano, 2011). In addition, condensins are also involved in decatenating dsDNA, DNA damage response, DNA repair, and cancer growth (Hirano, 2012). However, the detailed mechanisms of condensins in human nervous system diseases have not been sufficiently illustrated. To date, their trials aimed at investigating condensins related drugs for the treatment of nervous system diseases are lacking. In this regard, we can say that the biological function of condensin is still at an early stage of investigation and obviously requires more attention from researchers.

## Condensin and Nervous System Developmental Disorders

Microcephaly is a group of neurodevelopmental disorders characterized by a decrease in head circumference accompanied by a certain degree of non-progressive intellectual degradation (Neitzel et al., 2002). Patients with primary microcephaly, whose cells display unique cellular phenotypes, including premature chromosome condensation (PCC) in the G2 phase, present with intellectual disability. This group of patients shows a decrease in the size of the brain and a dramatic reduction in the size of the head (Neitzel et al., 2002; Darvish et al., 2010). Microcephalin/MCPH1 is one of the pathogenic genes of primary microcephaly caused by autosomal recessive inheritance (Jackson et al., 2002; Trimborn et al., 2004; Venkatesh and Suresh, 2014). It has been shown that it is primarily condensin II, not condensin I, that is involved in MCPH1-deficient microcephaly (Trimborn et al., 2006). This is mainly caused by condensin II locating to the nucleus from interphase through prophase and participates in an early stage of chromosome condensation within the prophase nucleus. This is consistent with PCC in MCPH1-deficient microcephaly patients. Moreover, condensin II needs to bind to chromatin at the right time and at the appropriate dosage to achieve the correct condensation of chromatin and MCPH1 is one of the factors that regulates this process (Trimborn et al., 2006; Wood et al., 2008; Yamashita et al., 2011). Furthermore, MCPH1 can bind with NCAPG2 and NCAPD3 subunits of condensin II through its intermediate domain and N-terminal BRCT domain, then regulate SMC2-NCAPH2 interface, to prevent condensin II from associating with DNA stably and reduce the formation of extruding loops (Wood et al., 2008; Yamashita et al., 2011; Houlard et al., 2021; Liu et al., 2021). Nevertheless, patients with MCPH1-deficient microcephaly lacked properly functioning MCPH1. As a result, condensin II loses normal inhibitory regulation of MCPH1, which makes condensin II combine with chromatin prematurely and more tightly. This further leads to PCC in the G2 phase of the cell cycle, which perturbs the program of gene expression supporting normal development of the brain (Trimborn et al., 2006; Yamashita et al., 2011). Microcephaly is thus formed. The above pathological process is the pathogenic model of MCPH1-deficient microcephaly recognized by many scholars nowadays.

There is another kind of microcephaly, which is mainly caused by the deletion or mutation of the gene encoding the condensin subunits. Here are some reports about condensin-deficient microcephaly: in 2010, Chen et al. (2010) reported a child with a chromosome 22q13 deletion (including NCAPH2 deletion), who showed intellectual disability, autism, epilepsy, and developmental delay. In the same year, Ji et al. (2010) reported two cases of Jacobsen syndrome (JBS) which is a haploinsufficiency syndrome caused by the deletion of part of the long arm of chromosome 11, including the deletion of NCAPD3. In that two cases, both patients presented with developmental delay, microcephaly, and facial deformity. In 2013, Perche et al. (2013) reported a case of 7qter deletion syndrome with NCAPG2 deletion, in which the patient is presented with microcephaly and intellectual disability. In 2016, Martin et al. (2016) found that the double allele mutation of NCAPD2, NCAPH, and NCAPD3 would lead to microcephaly. In 2019, Khan et al. (2019) reported two families of NCAPG2 recessive mutations and their clinical phenotypes, including neurodevelopmental defects and ocular abnormalities.

Further studies have shown that fibroblasts from these patients share the same genetic pathological changes, including excessive DNA/chromatin bridges (a kind of remaining connective structure of incomplete separation of chromosomes), lagging chromatin/chromosomes, micronuclei, and aneuploidy, in cells during mitosis (Martin et al., 2016; Khan et al., 2019). Interestingly, condensin I and condensin II are just what are needed to break down DNA/chromatin bridges (Charbin et al., 2014; Martin et al., 2016; Piskadlo et al., 2017). Therefore, once some subunit gene sites of condensin are mutated or deleted, such as NCAPD2, NCAPH, and NCAPD3, this will damage the function of condensin to decompose DNA/chromatin bridges. The above genetic pathological changes will occur during cell mitosis. These genetic pathological changes can also reduce cell proliferation and increase cell death in the cerebral cortex, providing a straightforward explanation for condensin-deficient microcephaly. So another pathogenic model of microcephaly was proposed that “condensinopathies” result in condensin-deficient microcephaly due to impaired DNA decatenation (Charbin et al., 2014; Martin et al., 2016; Piskadlo et al., 2017; Khan et al., 2019).

To sum up, there are two pathogenic models of microcephaly, MCPH1-deficient and condensin-deficient. Both of them indicate that maintaining dynamic stability, proper organization, correct shape, and ordered isolation of 3D genome is crucial for neurodevelopment (Davis et al., 2018; Ghosh and Meyer, 2021; Cummings and Rowley, 2022) in which condensins play an indispensable role. Many subunits of condensing are involved in the two pathogenic models. However, the specific role of each subunit is still unclear and needs further research. Related condensin subunits and symptoms of nervous system developmental disorders are summarized in Table 1.

**Table 1:** Subunits and symptoms of condensin associated with nervous system developmental disorders.

Condensin subunits involved in	Symptom manifestations
SMC2	Microcephaly
NCAPG2	Intellectual disability
NCAPD3	Autism
NCAPH2	Epilepsy
NCAPD2	Facial deformity
NCAPH	Brain structural abnormality
	Mental disorder
	Aphasia
	Ocular anomalies

## Condensin and Nervous System Tumors

Glioma is a kind of tumor that originated from glial cells and is the most common primary malignant tumor of the brain (Malzkorn and Reifenberger, 2016). Despite the etiology of glioma is not very clear till present, more and more studies have shown that the pathogenesis of glioma is related to the 3D genome structure in which condensins play a pivotal role (Phillips et al., 2020; Wang et al., 2021). Currently, studies have shown that the subunits of condensin, such as NCAPG, SMC4, and NCAPG2, have been reported to be involved in the glioma pathogenesis. Wherein, NCAPG could be positively related to CDCA2 (cell division cycle-associated protein 2) in glioma, and the over-expression of NCAPG may regulate the cell cycle and promote the proliferation, migration, and invasion of glioma cells (Liang et al., 2016; Jiang et al., 2022; Jin et al., 2022). Meanwhile, NCAPG overexpression can also increase the expression of MHCI and AMAD17 molecules, both of which are located on the tumor surface, thus, assisting in camouflaging the tumor and preventing NK cells from being activated in the immune microenvironment (Zheng et al., 2022). In addition, the knocking down of NCAPG can make the tumor cells stay in the G1 phase of the cell cycle. Unfortunately, there is still no research to clarify how NCAPG regulates the cell cycle and how to promote glioma progression by affecting the 3D genome structure of glioma. Further investigations of that are needed to be conducted to prove the regulative relationships.

Numerous studies have proved that SMC4 is involved in glioma molecular nosogenesis. The overexpression of this condensin subunit in glioma cells can increase their proliferative by accelerating the G1-S phase transition, and thus can promote migration, invasive, tumorigenicity, and the epithelial-mesenchymal transition (EMT) process of glioma cells (Jiang et al., 2017; You et al., 2021). According to recent researches, the above effects of SMC4 in glioma cells are exerted mainly by activating the TGF β/Smad pathway, along with Smad2/3 phosphorylation and Smad2/3 nuclear translocation (Jiang et al., 2017). The TGFβ/Smad pathway is a recognized signal pathway that promotes the progression of glioma, characterized by p-Smad2 nuclear translocation (Bruna et al., 2007). The p-Smad2 co-locates with DSB repair protein in the nucleus and participates in inducing DNA damage response (DDR; Hubackova et al., 2012; Wang et al., 2013). A hallmark of cancers is their genomic instability due to a propensity to accumulate DNA damage (O’Connor, 2015). Therefore, the tumor depends on DDR to maintain its survival (O’Connor, 2015; Bakhoum et al., 2017). The 3D genome structure needs to be appropriately changed to assist DDR (Yasuhara and Zou, 2021), and the Smc2/4 condensin complexes are needed in this process (Wu and Yu, 2012). In the SMC4 depleted glioma cells, the DNA damage foci increased significantly (Wang and Wu, 2021). SoSMC4 may participate in DDR directly or indirectly, to maintain genome stability and promote the malignant progression of glioma.

It has been shown that the overexpression of NCAPG2 could promote proliferation, migration, and invasion and regulate the cell cycle in glioblastoma cells. NCAPG2 regulates HBO1 phosphorylation and H4 histone acetylase activation, modulates activation of the Wnt/β-catenin pathway, increases the expression of MCM, and the binding of MCM protein to chromatin (Wu et al., 2021). MCM mainly participates in DNA replication by binding chromatin in the S phase, which is required by chromosome condensation mediated by condensin II (Sonneville et al., 2015). The surplus of MCMs can increase the robustness of genome duplication by restraining the speed at which eukaryotic cells replicate their DNA (Sedlackova et al., 2020). MCM can also participate in DDR (Drissi et al., 2018). SoNCAPG2 may also promote the malignant progression of glioma by maintaining genome stability.

Some studies have shown that, not only in glioma, condensins also play an important role in the occurrence and development of other kinds of nervous system tumors. Neuroblastoma is a developmental neoplasm of the autonomic nervous system that primarily affects young children (Fetahu and Taschner-Mandl, 2021). In neuroblastoma, MYCN gene amplification is related to the poor prognosis of patients (Tolbert and Matthay, 2018). The overexpression of SMC2, in neuroblastoma with MYCN gene amplification, can also promote tumor growth by regulating DDR (Murakami-Tonami et al., 2014). A typical teratoid/rhabdoid tumor (AT/RT) is a highly malignant central nervous system tumor predominantly occurring in infants and possibly also in older children and adults (Fruhwald et al., 2016). NCAPG is a potential oncogene of AT/RT, which is mainly involved in the cell cycle, DNA replication, and the p53 signaling pathway (Pan et al., 2020).

In short, NCAPG, SMC2/4, and NCAPG2, the subunits of condensins, play an important role in the malignant progression of nervous system tumors. There is a potential pathogenic model of nervous system tumors: because the tumor itself has genome instability and DNA damage, the tumor will compensate for over-expression of condensins. For one thing, condensins can directly participate in genome condensation to maintain chromosome stability; for another, condensins can indirectly maintain genome stability through the DDR pathway or MCM protein pathway (Ibarra et al., 2008). This can, rapidly and safely, promote the G1-S phase transition of the tumor, realize the effective proliferation of the tumor, and lay the foundation for the malignant progression of the tumor.

## Condensin and Alzheimer’s Disease

Alzheimer’s disease (AD) is a neurodegenerative disease with the hidden onset and progressive development. Clinically, it is characterized by memory disorder, aphasia, apraxia, agnosia, impairment of visual and spatial skills, executive dysfunction, personality and behavior changes, etc (Knopman et al., 2021). However, the etiology of AD is still unclear. It has been shown that DNA methylation, chromatin remodeling, and 3D genomic structural abnormalities are potential causes of AD (Berson et al., 2018). Studies have also shown that there is genetic variation related to AD in a wide area in chromosome12 (Pericak-Vance et al., 1997; Liang et al., 2006). Later, Lee et al. (2008) and Li et al. (2009) successively and accurately located NCAPD2 as a potential susceptibility gene of AD in chromosome 12p13. Unfortunately, there has been no research report on NCAPD2 and AD since then, and the relationship between them has become a mystery. Kobayashi et al. (2016), Shinagawa et al. (2016), and Chen et al. (2021) have successively confirmed that the level of NCAPH2 methylation in peripheral blood of AD patients is lower than that of normal people and NCAPH2 hypomethylation is significantly positively associated with the hippocampal volume in patients. Similarly, further relationship between NCAPH2 and AD remains to be revealed.

Moreover, we found that there are some potential relationships between condensins, cellular senescence, and AD. These may help us to reveal the roles of condensins in AD. At first, studies have shown that cellular senescence emerges as a pivotal player in the complex cellular landscape of AD, which could promote Aβ deposition, tau-dependent pathology, and cognitive decline (De Strooper and Karran, 2016; Bussian et al., 2018; Musi et al., 2018; Guerrero et al., 2021). Then in mouse models of neurodegeneration, clearance of senescent glial cells alleviates tau-dependent neurodegeneration and β-amyloid plaque size (Bussian et al., 2018; Guerrero et al., 2021). And ablation of senescent cells has been postulated as a promising therapeutic means to prevent or mitigate AD (Saez-Atienzar and Masliah, 2020). Meanwhile, senescent processes are accompanied by significant alterations in the 3D genome architecture, such as the formation of senescence-associated heterochromatic foci (SAHF) and the distension of centromeric satellites (Iwasaki et al., 2019). There are several models of cellular senescence and different cellular senescence models may have disparate and unique chromatin changes (Hernandez-Segura et al., 2018; Huang et al., 2019; Wang et al., 2020). Among them, oncogene-induced senescence (OIS) and replicative senescence (RS) are two common models of cellular senescence, in which condensins are involved (Wang et al., 2020). On the one hand, overexpression of condensin complex II subunit NCAPH2 induces SAHF formation in OIS and contributes to senescent processes, *via* reorganizing the 3D genome, reinforcing euchromatic A compartments, and upregulating transcription of senescence genes (Yokoyama et al., 2015; Iwasaki et al., 2019; Wang et al., 2020). On the other hand, condensin complex I subunit NCAPD2 promotes heterochromatin condensation and reorganization, and overexpression of the condensin complex subunit rescues RS-induced cellular senescence and heterochromatin destruction (Huang et al., 2019; Wang et al., 2020). Therefore, in humans, low expression of NCAPD2 and overexpression of NCAPH2 induce cellular senescence, and then cellular senescence promotes the formation and progress of AD.

In a word, NCAPD2 and NCAPH2 are inextricably linked with the occurrence and progress of AD, but current research is still at the level of gene sequencing. Based on previous literature, we proposed a possible pathogenic model: condensin I and condensin II may induce cellular senescence by regulating 3D chromatin architecture, then participate in the occurrence and progress of AD. The detailed and matured mechanism needs further in-depth research and verification.

## Summary

In summary, condensins play an indispensable role in the rational concentration and accurate separation of chromatin. The occurrence and progress of many nervous system diseases are inseparable from the condensation and the decondensation functions of condensins: excessive condensation and insufficient condensation of chromatin can both lead to microcephaly; compensatory enhanced chromatin condensation counteracts the tumor genome instability and promotes the survival and development of nervous system tumors; reorganizing the 3D chromatin architecture and upregulating transcription of senescence genes induce the formation and progress of AD. The discovery of these pathogenic mechanisms may change the understanding of related diseases and provide new ideas for clinical treatment.

It should be noted that condensins could be diversely regulated in the contexts of different cell activities, such as cell differentiation, gene mutation, genome instability, and others. Cell differentiation needs to change 3D chromatin architecture by regulating condensins to achieve selective gene expression and spatio-temporal specificity. When condensins are abnormally regulated by gene mutation and other cell activities, the malfunction of this protein complex will contribute to many diseases. Therefore, the functions of condensins of cells in different differentiation states or mutation loading states may be different. This may explain why different pathologies arise after the same condensin subunit is affected. Thus, we are inclined to believe that the change in the components of condensin ultimately exerts pathological effects through the regulation of 3D chromatin architecture by condensin complexes.

To sum up, in one way, the molecular structure of condensing and its biological function in mitosis have not been explained until recent years, and the mechanism of assisting linear DNA condensed into 3D chromatin architecture is still worth further research. In the second way, there are many subunits of condensin, and the functions of other subunits in different nervous system diseases need to be studied and revealed one by one. At present, research on condensin in various human diseases, especially in tumors, is in a period of rapid growth and is expected to become a research hotspot in the future.

## Author Contributions

XY provided the idea. SY contributed to the search and assessment of the available literature. DP mainly wrote the manuscript. All authors contributed to the article and approved the submitted version.

## Funding

This work was partially supported by the National Key Research and Development Program of China (No. 2018YFC0115603), the National Natural Science Foundation of China (No. 81872063), and the Clinical Research Program of Tianjin Medical University (No. 2018kylc001).
